# The Basic vs. Ability-to-Pay Approach: Evidence From China's Critical Illness Insurance on Whether Different Measurements of Catastrophic Health Expenditure Matter

**DOI:** 10.3389/fpubh.2021.646810

**Published:** 2021-03-18

**Authors:** Ying Zhang, Yongmei Guan, Ding Hu, Jacques Vanneste, Dongmei Zhu

**Affiliations:** ^1^School of Economics and Management, Southeast University, Nanjing, China; ^2^Business School, Nanjing University, Nanjing, China; ^3^Faculty of Business and Economics, University of Antwerp, Antwerp, Belgium

**Keywords:** catastrophic health expenditure, critical illness insurance, basic approach, ability-to-pay approach, China, I13, I18, G22

## Abstract

Alleviating catastrophic health expenditure (*CHE*) is one of the vital objectives of health systems, as defined by the World Health Organization. However, no consensus has yet been reached on the measurement of *CHE*. With the aim of further relieving the adverse effects of *CHE* and alleviating the problem of illness-caused poverty, the Critical Illness Insurance (*CII*) program has been operational in China since 2012. In order to verify whether the different measurements of *CHE* matter under China's *CII* program, we compare the two-layer *CII* models built by using the basic approach and the ability-to-pay (*ATP*) approach at a range of thresholds. Exploiting the latest China family panel studies dataset, we demonstrate that the basic approach is more effective in relieving *CHE* for all insured households, while the *ATP* approach works better in reducing the severity of *CHE* in households facing it. These findings have meaningful implications for policymaking. The *CII* program should be promoted widely as a supplement to the current Social Basic Medical Insurance system. To improve the *CII* program's effectiveness, it should be based on the basic approach, and the threshold used to measure *CHE* should be determined by the goal pursued by the program.

## Introduction

As a kind of financial shock, catastrophic health expenditure (*CHE*) is a critical contributor to income and expenditure uncertainty, which in turn affect social welfare around the world (1–3). It is often justified as a way to create heavy financial burden for the sake of necessary health care and forces people to suffer from loss of income owing to their reduced labor supply and fall in productivity (4, 5); this significantly lowers the living standards of residents and pushes households below, or further below, the poverty line (6, 7).

However, no consensus has yet been reached on the measurement of *CHE*. Two approaches—the basic and ability-to-pay (*ATP*)—have been proposed (8). The basic approach defines *CHE* as spending for health care that exceeds a certain level of the patient's total income, consumption, or expenditure. The *ATP* approach defines *CHE* as the amount by which health spending exceeds the threshold of a household's ability to pay for health care. In addition, there is no agreement among health economists on the threshold of household expenditure (9). The basic approach usually uses 10% as the threshold value (10), whereas the *ATP* approach commonly adopts 40% (11). Some studies also compare a range of thresholds—typically 10–40%—to measure *CHE* (8). According to the previous literature, these two approaches have their own strengths and drawbacks. For instance, the basic approach defines as easy to understand, requires no further calculation, and does not depend on household allocation decisions; the *ATP* approach can make up for the shortcoming in the basic approach—its inability to distinguish between the poor and the rich (12).

Therefore, the present study intends to investigate whether using different approaches to measuring *CHE* affects China's Critical Illness Insurance (*CII*) program. Specifically, there are two methodological issues to consider. The first is to identify household resources that are available for health spending. The second is to determine the threshold used to identify health expenditures as catastrophic. Thus, we construct two-layer *CII* models based on the basic and *ATP* approaches and adopt a range of thresholds to measure *CHE* through two approaches. The empirical analysis is performed with household-level data from the latest China family panel study dataset, which is a national social survey project that includes detailed survey data on counties, households, and family members in 25 provinces and autonomous regions. Our study intends to expand the existing research by revealing that different measurements of *CHE* can lead to different performances in China's *CII* program. Further, as the second-largest economy and the most populous country in the world, China has established a *CII* program that covers a high percentage of *CHE*; thus, the *CHE* measurement approach used in China can provide reliable and worthwhile insights for countries around the world.

The remainder of the paper is organized as follows. Section Institutional Background and Comparison of Approaches provides a detailed introduction to China's *CII* program and compares the basic approach with the *ATP* approach. Section Two-Layer CII Models Based on the Two Approaches constructs two-layer *CII* models based on these two approaches. Section Empirical Results and Comparison makes the empirical comparison. Section Concluding Remarks concludes.

## Institutional Background and Comparison of Approaches

### Development of the Critical Illness Insurance Program in China

**Figure 1:**
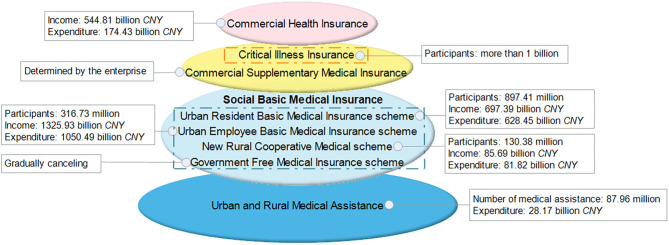
China's multilevel medical insurance system and its operation (2018).

Since 1998, China has gradually established a multilevel medical insurance system (see Figure 1), of which the Social Basic Medical Insurance (*SBMI*) system is an essential component. The *SBMI* system consists of the following four schemes: the Government Free Medical Insurance (*GFMI*); the Urban Employee Basic Medical Insurance (*UEBMI*); the Urban Resident Basic Medical Insurance (*URBMI*); and the New Rural Cooperative Medical (*NRCM*). By the end of 2018, more than 95% of the population has been covered by the *SBMI* system, whose total expenditure accounted for 30.36% of the total medical expenses (13). However, the out-of-pocket (*OOP*) expenses in China remained high and, in some instances, catastrophic; they accounted for 28.77% of the total health expenditure in 2017.

With the aim of further reducing *CHE* and alleviating the problem of illness-caused poverty, the *CII* program—a supplement to the *SBMI* system—was launched in 2012 and fully implemented in 2015.

#### Coverage of the *CII*

The *CII* program covers the enrollees of both the *URBMI* scheme and the *NRCM* scheme. The two schemes, covering urban unemployed and self-employed residents and rural residents, are collectively referred to as the Social Resident Basic Medical Insurance (*SRBMI*). After 6 years of operations, the coverage of the *CII* program has greatly improved. By the end of 2017, the *CII* program had covered 1.06 billion residents.

The *CII* program is a medical program that compensates its members for *CHE* owing to critical illnesses that exceed the cap line of *SRBMI*. There are some differences in the scope of diseases reimbursed by *CII* for urban and rural residents. For urban residents, the diseases mainly include malignant tumor chemotherapy (including endocrine-specific antitumor treatment), malignant tumor radiotherapy, isotope antitumor treatment, interventional antitumor treatment, traditional Chinese medicine antitumor treatment, hemodialysis, and dialysis for severe uremia, anti-rejection treatment after kidney transplantation, and mental illness treatment (including schizophrenia, moderate to severe depression, mania, obsessive–compulsive disorder, mental retardation accompanied by mental disorder, epilepsy associated with mental disorder, and paranoid psychosis).

For rural residents, the diseases covered include gastric cancer, esophageal cancer, colon cancer, rectal cancer, lung cancer, liver cancer, breast cancer, cervical cancer, end-stage kidney disease, childhood congenital heart disease, childhood leukemia, childhood lymphoma, cataract, pneumoconiosis, neuroblastoma, osteosarcoma, hemophilia, thalassemia, cleft lip and palate, hypospadias, multidrug-resistant tuberculosis, stroke, chronic obstructive emphysema, and AIDS-related opportunistic infections.

However, in some provinces the scope of *CII* compensation is defined by the level of medical expenses, rather than disease type. For example, in Beijing, the deductible under *CII* is the annual per capita disposable income of rural residents in the previous year. When an individual's *OOP* expenses exceed the deductible, it means that the individual has suffered from critical illnesses.

The specific reimbursement rate of the *CII* program (i.e., 55% in Shanghai and 60% in Hebei) is determined by the local government; however, the central government has mandated that the rate should be no <50%. By the end of 2017, the overall reimbursement ratio of *CII* had reached 70%, far exceeding the target of “not <50%” (14).

#### Financing Patterns of the *CII*

Currently, the *CII* program is financed by the *SRBMI* Fund. If there is sufficient surplus in the *SRBMI* fund, part of the surplus is to be used as *CII* fund; otherwise, part of the *SRBMI* fund raised in that year will be set aside for the *CII* fund. The specific financing patterns can be divided into two types. The first allocates a certain percentage—the most common is 5% and has been implemented in Beijing, Fujian, Guangdong, Guizhou, Hubei, Hunan, Inner Mongolia, Jiangxi, and Jiangsu—of the *SRBMI* fund to the *CII* fund. The other type uses a certain amount—ranging from CNY 28 to CNY 80 per person—from the *SRBMI*. Most provinces, municipalities, and autonomous regions in China adopt the first pattern, which is supplemented by the second pattern. In general, no matter what kind of financing pattern is adopted, the funding standard is low, and the funding source is not sustainable (15).

According to the policy requirements, the *CII* program should be implemented at least at the municipal level, while provincial-level coordination is encouraged to improve the ability to diversified risks. At present, only nine out of the 34 provinces, including the four direct-controlled municipalities, that is, Beijing, Tianjin, Shanghai, and Chongqing and five other administrative units, that is, Jilin, Gansu, Qinghai, Hainan, and Tibet, have achieved provincial-level coordination for the *CII* program. All the other regions have municipal-level coordination.

#### Problems of the *CII*

The current *CII* program needs further improvement. On the one hand, the coverage of the *CII* program should be increased to reduce *OOP* expenses. The *OOP* expenses in China accounted for 28.77% of the total health expenditure in 2017—a figure that is much higher than the world average of 18.15% in 2015. On the other hand, the pricing mechanism needs to be converted from fixed premium [fixed amount or a percentage of the *SRBMI* fund] to actuarial pricing to maintain the fund's sustainable performance.

### The Basic vs. Ability-to-Pay Approaches

#### The Definitions

*CHE* is an indicator reflecting the effectiveness of the financial protection that a health insurance policy could provide for its members, and investigating the extent of *CHE* is the first step to develop appropriate policy responses (11). In previous studies, the basic and *ATP* are two most commonly used approaches for measuring *CHE*.

The basic approach defines *CHE* as spending for health care that exceeds a certain level of a household's entire budget, that is, its total income (16–18) consumption (19, 20), or expenditure (21–23). The household budget is defined as the value of consumption in low- and middle-income countries, but in the high-income countries, it is given by the expenditure on goods and services (24). In empirical studies, the expenditure method takes precedence (25). If total household expenditure is not available, income or consumption can be used as its proxy variable (25).

The *ATP* approach defines *CHE* as health spending that exceeds the threshold of a household's ability to pay for health care (9, 11, 22, 26). According to different understandings of household's ability to pay, the *ATP* approach can be further divided into three methods—actual food spending, partial normative food spending, and normative spending on food, housing, and utilities (25). The main difference among the three methods is the basic need that is subtracted from household total expenditure to calculate *CHE*.

#### The Comparison

There is much academic debate on the applicability and effectiveness of the basic and *ATP* approaches. Both approaches have their own advantages and limitations, and neither approach is universally applicable. Therefore, the choice of approach requires specific consideration of the problem studied and the availability of data. A comparison of the two approaches is given below.

The basic approach, which uses household total income, consumption, or expenditure as the denominator for calculation of *CHE*, has the virtue of simplicity. A further advantage of this approach is that it is not dependent on household allocation decisions across consumption items (12). However, it has some limitations. First, it fails to distinguish between poor households that just manage to meet subsistence needs and rich households that enjoy some latitude in spending (12). Second, if households finance a substantial share of their health payments through coping strategies, the gross expenditure on *OOP* payments is not equal to the resources available for non-medical consumption; thus, in this case, poverty will be underestimated (8). Finally, the measurement of *CHE* and impoverishment that ignores the personal financing means can mislead in terms of the consequences of high *OOP* expenses. It can not only exaggerate the risk to current consumption and *CHE* but also overlook the long-term burden of health payments (3).

The *ATP* approach can address the first limitation of the basic approach effectively. Because it assumes that the poor households would spend a much higher proportion of available resources on necessary items (commonly food, rent, and utilities) than the rich households, it defines household resources as being net of such spending, that is, actual food spending or a standard amount representing the subsistence spending. Nevertheless, the *ATP* approach has its own drawbacks. First, it is unhelpful in determining the extent to which *OOP* expenses eat into resources required for necessities (27). Besides, the usefulness of the way used to measure the ability to pay remains doubtful. If the household can borrow or save to finance their *OOP* medical expenses, its ability to pay should be measured by consumption; otherwise, it needs to be measured by income (27, 28). However, it is often the case that the precise income data is unavailable in low- and middle-income countries, so there is no option but to use household consumption; this can lead to a misestimation of the incidence of *CHE* (28).

#### The Threshold

The threshold level used to identify *CHE* in empirical papers is arbitrary. For the Sustainable Development Goals (*SDGs*), *CHE* is measured by the basic approach, in which both 10% (10, 21, 29) and 25% of the budget are commonly used to determine it. Among studies using total consumption, 10% is more common and is used in 41% of the literature; 5, 20, and 25% of consumption are less common and account for 13, 12, and 6%, respectively, of the literature (28). The *ATP* approach commonly adopts 25% (25) or 40% (30, 31) as the threshold value. Besides, a range of thresholds, typically extending from 10 to 40%, are used to compare the difference in *CHE* between the basic approach (32, 33) and the *ATP* approach (34–37). Scholars also compare the performances of these two approaches at different thresholds (8, 38–40). The existing literature shows that the incidence and intensity of *CHE* and the poverty impact decrease when the threshold rises (32, 34, 36). Some papers arrive at the conclusion that the incidence of *CHE* in the basic approach at the 10 % threshold is higher than that in the *ATP* approach at the 40% threshold (24, 38).

## Two-Layer *CII* Models Based on the Two Approaches

### The Models

The two-layer *CII* models (41, 42) are constructed to act as a bridge between the *SRBMI* and private health insurance (*PHI*). In these models, we assume that the *CII* covers all the enrollees of *SRBMI* and compensates inpatient medical expenses partially by the choice of the deductible, compensation ratio, and cap line.

#### The First Layer

The first layer *CII* model uses the reinsurance technique to compensate the excessive cumulative annual inpatient medical expense above the deductible *CD*, but within the cap line *TL*_1_, and the compensation ratio is (1 − β_1_).

When *CD* < *x*_*i*_ ≤ *TL*_1_, the *OOP* expense can be expressed as:

(1) $$\begin{array}{l} OOP_{i}=\beta_{1}\left(x_{i}-CD\right)+\alpha CD \end{array}$$

Here, *x*_*i*_ is the cumulative annual medical expense of household *i*, and the compensation ratio in *SRBMI* is (1 − α).

#### The Second Layer

The second layer *CII* model uses the coinsurance technique to partially compensate for the *OOP* expense. The cap line and compensation ratio are *TL*_2_ and (1 − β_2_), respectively.

When *TL*_1_ < *x*_*i*_ ≤ *TL*_2_, the *OOP* expense can be expressed as:

(2) $$\begin{array}{l} OOP_{i}\text{ = }\beta_{2}\left[\beta_{1}\left(TL_{1}-CD\right)+\alpha CD+x_{i}-TL_{1}\right] \end{array}$$

### The Indicators

#### Deductible

The deductibles for the *CII* models in the basic approach at 10 and 25% thresholds are 0.1*y*_1_ and 0.25*y*_1_, respectively, while in the *ATP* approach the deductibles at the 25 and 40% thresholds are 0.25*y*_2_ and 0.4*y*_2_, respectively. Here, *y*_1_ represents the household annual total expense and *y*_2_ represents the household annual non-food expense.

#### Cap Line

The cap line of the first layer and second layer can be expressed as follows:

(3) $$\begin{array}{l} TL_{1}=CD+\left(1-\alpha \right)CD/\beta_{1} \end{array}$$

(4) $$TL_{2}=2F^{-1}\left(99\%\right)$$

(4) Here, *F*(•) is the cumulative distribution function of household total expense or household non-food expense.

#### Compensation Ratio

The purpose of the *CII* program is to alleviate *CHE*. Based on this objective, the objective function can be expressed as.

(5) $$\begin{array}{l} \text{min }J_{i}=\sum_{1}^{n}[\beta_{1}(x_{i}-CD)+\alpha \times CD-CD{]}^{2}I(CD<x_{j}\leq b\times y_{i})\\ +\sum_{1}^{n}\{\beta_{2}[\beta_{1}(b\times y_{i}-CD)+\alpha CD+x_{i}-b\times y_{i}]-CD{\}}^{2}I(b\times y_{i}<x_{j}\leq TL_{2}) \end{array}$$

Here, *I*(•) equals to 1 when the inequality condition in the parentheses is satisfied, and 0 otherwise. The parameter *b*, which is related to the threshold used, satisfies the following relationship.

(6) $$\begin{array}{l} b=Threshold+\left(1-\alpha \right)\times Threshold/\beta_{1} \end{array}$$

**Figure 2:**
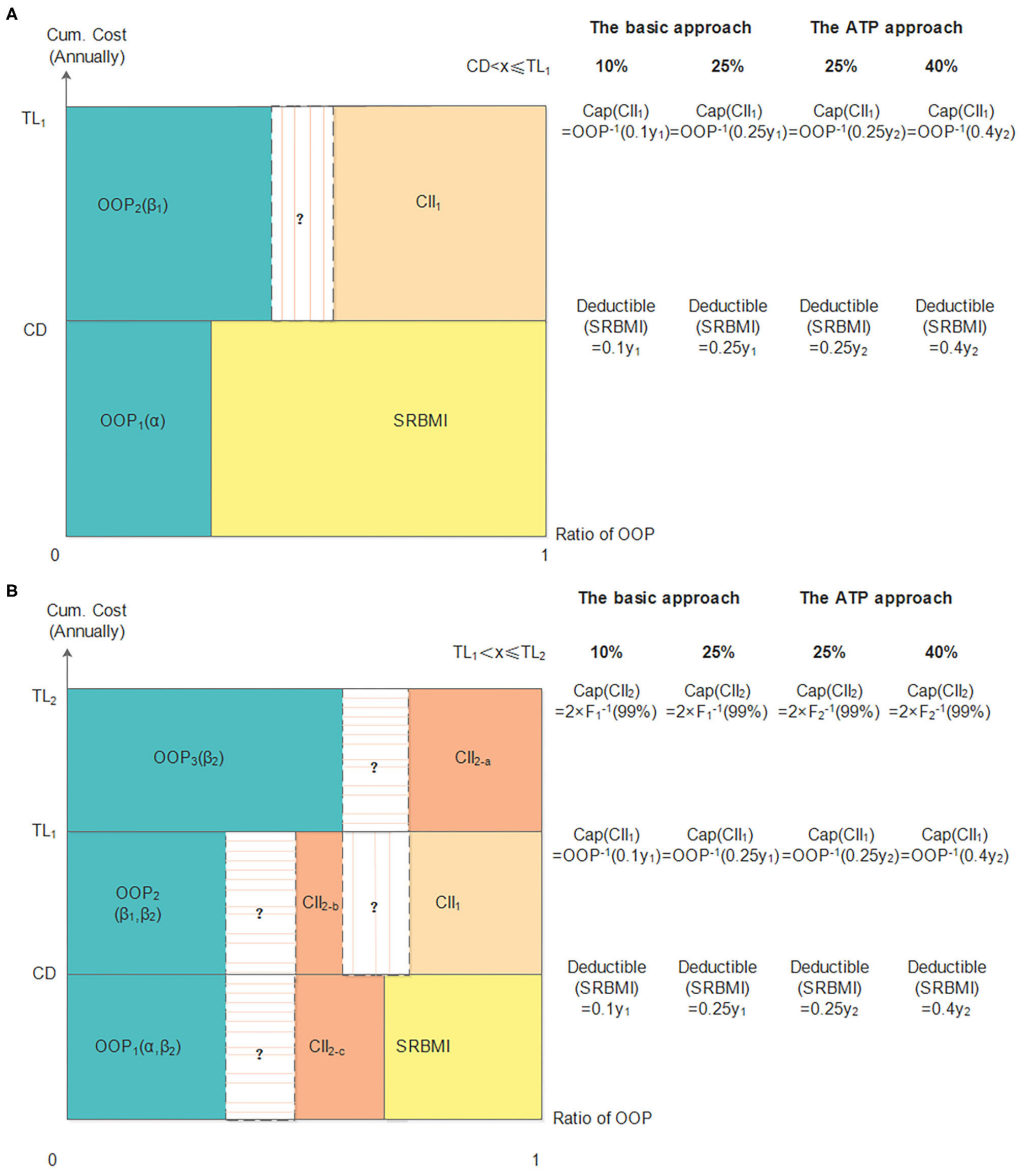
The two-layer *CII* models in the basic and *ATP* approaches. **(A)** shows the first layer *CII* model and panel **(B)** shows the two-layer *CII* model.

Detailed indicators of the *CII* models are shown in Figure 2.

### The Performance Evaluation

#### Incidence

The incidence is the ratio of insured households whose *OOP* expenses exceed the *CHE*. The number of insured households whose *OOP* expenses exceed the *CHE* is denoted as *e*, and the total number of insured households is denoted as *E*.

(7) $$\begin{array}{l} I=P\left(OOP_{i}\geq CD_{i}\right)=\frac{e}{E}\times 100\% \end{array}$$

#### Severity

Overall severity (*S*) is the average surplus of *OOP* expenses exceeding the *CHE* of all insured households. In order to measure the severity in households that incurred catastrophic expenses, regional severity (*S*_*reg*_) is proposed. It is the average value by which the *OOP* expenses exceed the *CHE* of those who suffer from *it*. Further, considering that the severity of household *CHE* can be affected by the number of family members, average severity (*S*_*ave*_) is adopted. It is the per capita surplus of *OOP* expenses beyond the *CHE* for all insured households. Here, *N* is the total number of household residents.

(8) $$\begin{array}{l} S\text{=}\frac{1}{E}\sum \left(OOP_{i}-CD_{i}\right) \end{array}$$

(9) $$\begin{array}{l} S_{reg}\text{=}\frac{1}{e}\sum \left(OOP_{i}-CD_{i}\right) \end{array}$$

(10) $$\begin{array}{l} S_{ave}\text{=}\frac{1}{N}\sum \left(OOP_{i}-CD_{i}\right) \end{array}$$

### Premium Setting

Assume that the probability of a member of a family being hospitalized is *P*(*h* = 1). The conditional probability distribution function can be expressed as follows:

(11) $$\begin{array}{l} P\left(h=1\right)=P\left(X_{1}B_{1}>\xi \right)=1-F_{1}\left(X_{1}B_{1}\right) \end{array}$$

Here, *X*_1_ represents the factors that affect *P*(*h* = 1), and *B*_1_ is the coefficient vector of *X*_1_. In this paper, the multivariate logit model is adopted to predict the probability of a member of a family being hospitalized.

*CHE* often exhibits the characteristics of an event with a low probability, but causing a large loss, and the distribution function is typically right-skewed and fat-tailed. Therefore, the lognormal distribution shown in Equation (12) can be adopted to fit high inpatient medical expenses. Assuming that household inpatient medical expenses and household total expense or non-food expense in the previous year are independent, the Weibull distribution (43) can be used to fit a household's total expense and its non-food expense. The joint probability density function is expressed in Equation (13).

(12) $$\begin{array}{l} \ln\;x\text{=}X_{2}B_{2}+\varepsilon \end{array}$$

(13) $$\begin{array}{l} f\left(x,y\right)=\frac{1}{\sqrt{2\pi }\sigma x}\frac{\alpha }{\beta }{\text{(}\frac{y}{\beta }\text{)}}^{\alpha -1}{\text{e}}^{-\frac{{\left(\ln\;x-X_{2}B_{2}\right)}^{2}}{2\sigma^{2}}-{\left(\frac{y}{\beta }\right)}^{\alpha }} \end{array}$$

Here, *X*_2_ represents the factors that affect household inpatient medical expenses, and *B*_2_ is the coefficient vector of *X*_2_.

In the first layer, the expected loss of *CII* can be expressed as:

(14) $$\begin{array}{l} EZ_{1}\text{=}\iint_{\begin{array}{c} 0<y\leq c\\ CD<x\leq by \end{array}}(1-\beta_{1})(x-CD)f(x,y)dxdy\\ +\iint_{\begin{array}{c} 0<y\leq c\\ x>by \end{array}}(1-\beta_{1})(by-CD)f(x,y)dxdy \end{array}$$

Considering that it is difficult to obtain medical expense data that fully satisfy the actuarial requirements, we add a risk loading that is 30%^1^ of the risk premium. Further, to attract commercial health insurers, the additional premium ratio is set at 15%.^2^. The pure premium and gross premium can be calculated as follows.

(15) $$\begin{array}{l} P_{pure1}=EZ_{1}\times P\left(h=1|X_{2}\right)/P\left(0<y\leq TL_{2}/b\right) \end{array}$$

(16) $$\begin{array}{l} P_{gross1}=P_{pure1}\times \left(1+30\%\right)/\left(1-15\%\right) \end{array}$$

In the second layer, the expected loss can be expressed as Equation (17).

(17) $$\begin{array}{l} EZ_{2}\text{=}\iint_{\begin{array}{c} 0<y\leq c\\ CD<x\leq by \end{array}}(1-\beta_{1})(x-CD)f(x,y)dxdy\\ +\iint_{\begin{array}{c} 0<y\leq c\\ by<x\leq TL_{2} \end{array}}(1-\beta_{2})[\alpha \times CD+\beta_{1}(by-CD)+x-by]f(x,y)dxdy\\ +\iint_{\begin{array}{c} 0<y\leq c\\ x>TL_{2} \end{array}}(1-\beta_{2})[\alpha \times CD+\beta_{1}(by-CD)+TL_{2}-by]f(x,y)dxdy \end{array}$$

We apply a risk loading of 40%^3^ of the pure premium, and the additional premium ratio is set at 15%. The pure premium and gross premium can be calculated as follows^4^:

(18) $$\begin{array}{l} P_{pure2}=EZ_{2}\times P\left(h=1|X_{2}\right)/P\left(0<y\leq TL_{2}/b\right) \end{array}$$

(19) $$\begin{array}{l} P_{gross2}=P_{pure2}\times \left(1+40\%\right)/\left(1-15\%\right) \end{array}$$

### The Fund Balance

The balance of the *CII* fund is measured by the difference between the fund's income and expenditure. The income and expenditure are expressed in Equation (20). Here, *EXP*_*i*_ is the reimbursement paid to household *i*.

(20) $$\begin{array}{l} I\text{ncome}=P_{gross}\times N \end{array}$$

(21) $$\begin{array}{l} Expenditure=\sum EXP_{i} \end{array}$$

## Empirical Results and Comparison

### Data Description

We use household-level data from the latest *CFPS* to test the performances of the *CII* models by using the basic and *ATP* approaches. The database is updated every 2 years, and after deleting missing data, our samples in 2010, 2012, 2014, and 2016 cover 7,579, 7,685, 10,080, and 7,056 households, respectively. The number of households that had inpatient experiences in 2010, 2012, 2014, and 2016 is 1,315, 1,842, 2,326, and 2,154, respectively. The definitions of the variables and the data descriptions are listed in the Appendix.

Our process of empirical analysis process is as follows. First, the samples in 2014 are used to fit household hospitalization probability and inpatient medical expense in 2016. Specifically, the multivariate logit model is adopted to predict the probability that a member of a family is hospitalized, and the ordinary least square (*OLS*) model is used to predict the conditional probability distribution function of household inpatient medical expenses. Then, by combining the coefficients in Table 5 in the Appendix and the linear trend of the variables between 2010 and 2014, the hospitalization probability and inpatient medical expense in 2016 can be predicted. After that, assuming that the compensation ratio of *SRBMI* in China is 0.7^5^, we can calculate the indicators of the *CII* models in the two approaches using the data from the *CFPS* (2012). At last, the performances of the *CII* models and the balance performances of the *CII* fund in the two approaches can be simulated using the data from *CFPS* (2016).

Table 1 shows that the hospitalization probability in 2016 is 24%, and the mean lognormal inpatient medical expense is CNY 8.89.

**Table 1:** The predicted parameters (2016).

**P(h=1|X_**1**_)**	**Lognormal distribution**	
	**Mean**	**Standard error**
0.24	8.89	1.18

### Threshold of *CHE*

Table 2 shows the household cumulative total expense and non-food expense. The average and 99th percentile of household total expense are CNY 42,027.97 and CNY 250,000, respectively. Thus, in the basic approach, the mean value of the household *CHE* at the 10 and 25% threshold are CNY 4,202.80 and CNY 10,506.99, respectively, and the cap amount of the second layer (*TL*_2_) is CNY 500,000. The average and 99th percentile of household non-food expense is CNY 27,305.52 and CNY 210,540, respectively. Accordingly, the mean value of household *CHE* at the 25 and 40% threshold in the *ATP* approach are CNY 6,826.38 and CNY 10,922.21, respectively, and *TL*_2_ in this approach is CNY 421,080.

**Table 2:** Household cumulative total expenses and non-food expenses (2014).

	**Total expenses**	**Non-food expenses**		**Total expenses**	**Non-food expenses**
1%	2,500	0			
5%	5,000	1,080			
10%	9,000	2,300			
25%	16,150	6,000			
50%	30,000	14,600	Obs.	10,080	10,080
75%	50,000	29,472	Mean	42,027.97	27,305.52
90%	80,000	54,000	Std. Dev.	69,839.95	66,401.83
95%	106,800	84,000	Skewness	20.18	22.71
99%	250,000	210,540	Kurtosis	713.74	845.93

### The Basic Approach's Performance

#### The Indicators

**Figure 3:**
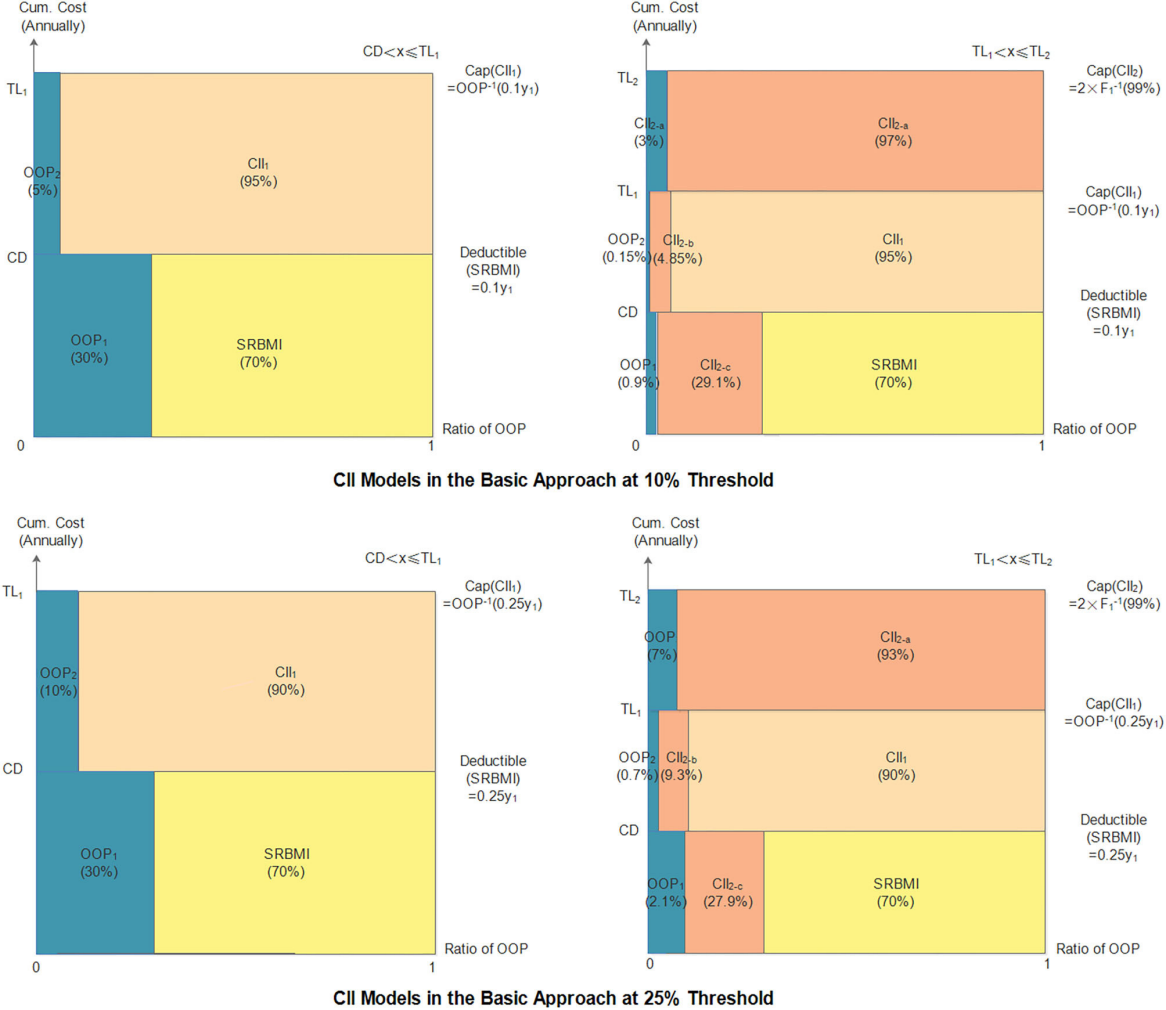
*CII* models in the basic approach.

The indicators of the *CII* models in the basic approach are shown in Table 3 and Figure 3. The two thresholds used in this approach are 10 and 25%. The deductible and the cap amount of the first layer at the 25% threshold are higher than those at the 10% threshold, and the cap amount of the second layer at both the thresholds is CNY 500,000. Accordingly, the compensation ratios (1–β_1_) and (1–β_2_), at the 25% threshold, are lower than those at the 10% threshold. Then, the final household cumulative *OOP* payments under the model can be calculated as shown in Table 4. When cumulative annual inpatient expenses are below *TL*_1_, *OOP* expenses are positively correlated with household total expenses; otherwise, they are negatively correlated.

**Table 3:** Indicators of the *CII* model in the basic approach.

**Threshold**	***CD***	**TL_1_**	**TL_2_**	**1-β_1_**	**1-β_2_**
10%	0.1*y*_1_	1.5*y*_1_	500,000	0.95	0.97
25%	0.25*y*_1_	2*y*_1_	500,000	0.9	0.93

**Table 4:** Household cumulative *OOP* expenses in the basic approach.

**Threshold**	**Cumulative annual**	***OOP* expenses**
	**inpatient expenses**	
10%	0.1y_1i_ < *x*_*i*_ ≤ 1.5*y*_1*i*_	0.05*x*_*i*_ + 0.03*y*_1*i*_
	1.5y_1*i*_ < *x*_*i*_ ≤ 500, 000	0.03*x*_*i*_ − 0.04*y*_1*i*_
	*x*_*i*_ > 500, 000	*x*_*i*_ − 0.04*y*_1*i*_ − 485, 000.00
25%	0.25y_1*i*_ < *x*_*i*_ ≤ 2*y*_1*i*_	0.10*x*_*i*_ + 0.05*y*_1*i*_
	2y_1*i*_ < *x*_*i*_ ≤ 500, 000	0.07*x*_*i*_ − 0.12*y*_1*i*_
	*x*_*i*_ > 500, 000	*x*_*i*_ − 0.12*y*_1*i*_ − 465, 000.00

#### The Performance Evaluation of the *CII* Model

As can be seen in Table 5, before any reimbursement, the incidence of *CHE* at the 10 and 25% thresholds is 23.57 and 15.32%, and the overall severity at the two thresholds is CNY 3,901.82 and CNY 3,068.25, respectively. In the two-layer *CII* models, the four indicators are all reduced to a much lower level. The incidence, overall severity, average severity, and regional severity at the 10% threshold are 0.85%, CNY 22.16, CNY 5.72, and CNY 2,605.56, respectively, and at the 25% threshold, they are 0.72%, CNY 21.26, CNY 5.49, and CNY 2,941.24, respectively. These results demonstrate that both the two-layer *CII* models can effectively reduce the incidence and severity of *CHE*. In addition, it is noticed that all indicators, except for regional severity, at the 10% threshold are higher than those at the 25% threshold; this means that the model at the 10% threshold is more effective in alleviating *CHE* for households suffering from it.

**Table 5:** Performance evaluation of the *CII* model in the basic approach.

	**Indicator**	**10%**	**25%**
Without any	*OOP*>*CHE*	1,663	1,081
insurance	incidence	23.57%	15.32%
	Overall severity	3,901.82	3,068.25
	Average severity	1,007.58	792.33
	Regional severity	16,555.17	20,027.34
*SRBMI*	*OOP*>*CHE*	1,311	763
	Incidence	18.58%	10.81%
	Overall severity	3,451.89	2,473.22
	Average severity	891.40	638.67
	Regional severity	18,578.62	22,871.65
*CII*_1_	*OOP*>*CHE*	240	181
	Incidence	3.40%	2.57%
	Overall severity	1,132.93	876.52
	Average severity	292.56	226.35
	Regional severity	33,308.16	34,169.82
*CII*_1_&*CII*_2_	*OOP*>*CHE*	60	51
	Incidence	0.85%	0.72%
	Overall severity	22.16	21.26
	Average severity	5.72	5.49
	Regional severity	2,605.56	2,941.24

#### The Premium Setting in the *CII* Model

The parameters of the probability distribution function of household total expense in 2012 can be calculated *via* maximum likelihood estimation (*MLE*). The scale and shape parameters are 42,985.86 and 1.05, respectively. Given the simplicity of calculation, we assume that household inpatient medical expenses and household total expenses in the previous year are mutually independent. The joint probability density function can be expressed as shown in Equation (22). Then, the pricing of the *CII* models can be simulated according to the premium calculation formulas of *Pricing I* and *Pricing II*. As can be seen in Table 6, both the pure and gross premiums in the two-layer *CII* models at the 10% threshold are higher than those at the 25% threshold.

(22) $$\begin{array}{l} f\left(x,y_{\text{2}}\right)=\frac{1}{\sqrt{2\pi }\times 1.\text{18}\times x}\times \frac{\text{1.05}}{\text{42,985.86}}\\ \times {\left(\frac{y_{\text{1}}}{\text{42,985.86}}\right)}^{\text{0.05}}e^{-\frac{{\left(\ln\;x-8.\text{89}\right)}^{2}}{2\times 1.\text{1}{\text{8}}^{2}}-{\left(\frac{y_{\text{1}}}{\text{42,985.86}}\right)}^{\text{1.05}}} \end{array}$$

**Table 6:** The *CII* pricing simulation in the basic approach.

**Threshold**	***CII* pricing**	**Pure premium per capita**	**Gross premium per capita**
10%	*Pricing I*	470.68	719.87
	*Pricing II*	566.77	933.51
25%	*Pricing I*	343.11	524.76
	*Pricing II*	426.94	703.20

#### The Fund Balance in the *CII* Model

We assume that all residents paid *Pricing I* or *Pricing II* in 2014; thus, the number of insured individuals is 37,984. As can be seen in Table 7, regardless of the pricing, the *CII* fund is fairly balanced and can have a slight surplus. Thus, the pricing of the proposed *CII* design can achieve the desired financial sustainability.

**Table 7:** The fund balance of the *CII* in the basic approach.

**Fund income**			**Fund expenditure**		
***Pricing I***	**10%**	**25%**		**10%**	**25%**
The number of the insured individuals	37,984	37,984	*CII* expenditure	18,537,148	13,871,085
The gross premium per capita	719.87	524.76	The insurance operation management expenses	2,734,356	1,993,239
The total income	27,343,560	19,932,393	The total expenditure	21,271,504	15,864,324
***Pricing II***					
The number of the insured individuals	37,984	37,984	*CII* expenditure	26,637,228	20,345,575
The gross premium per capita	933.51	703.20	The insurance operation management expenses	3,545,840	2,671,044
The total income	35,458,404	26,710,442	The total expenditure	30,183,069	23,016,619

### The *ATP* Approach's Performance

#### The Indicators

**Figure 4:**
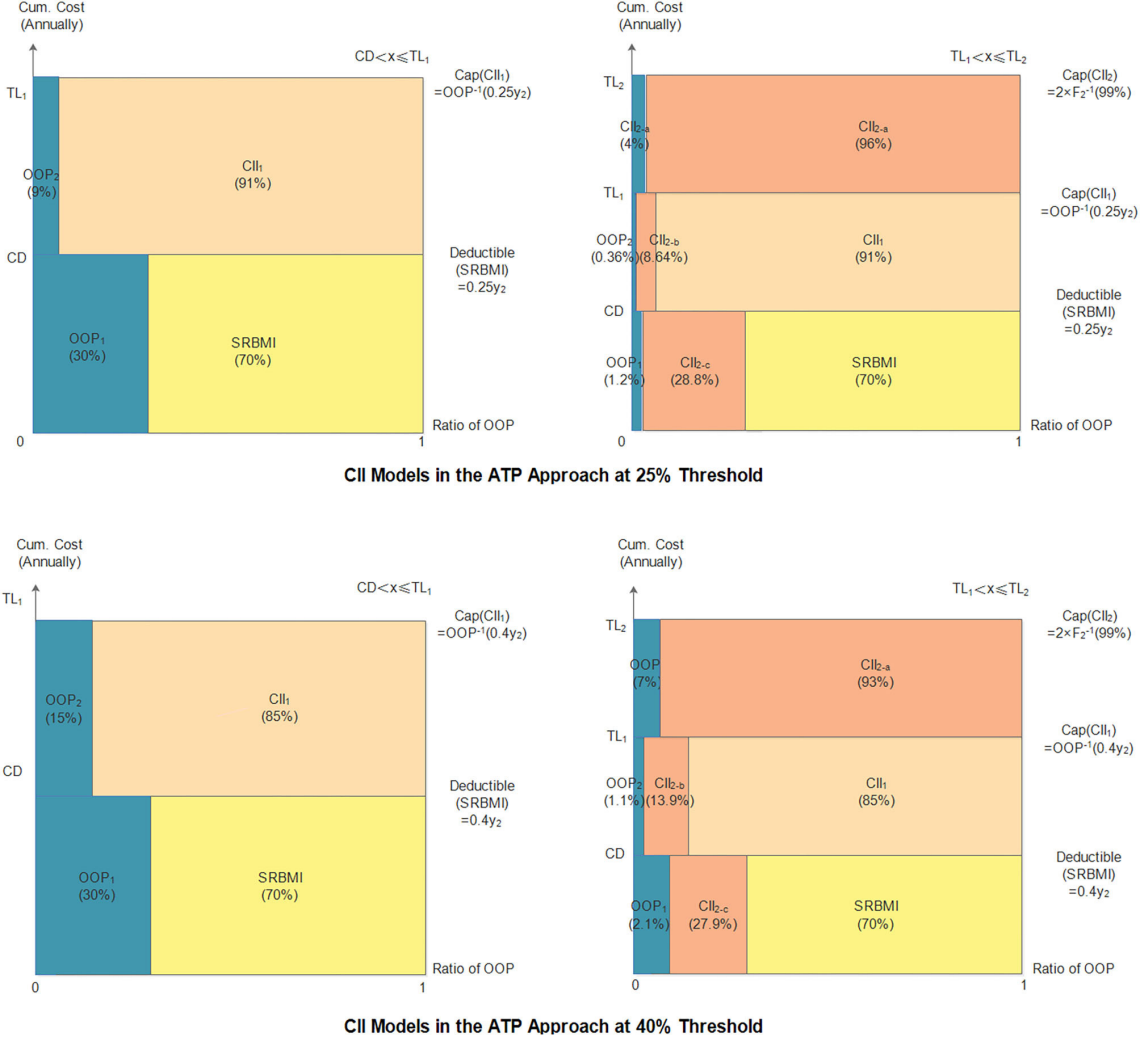
*CII* models in the *ATP* approach.

The indicators of the *CII* models in the *ATP* approach are shown in Table 8 and Figure 4. The two thresholds used in this approach are 25 and 40%. As in the basic approach, the deductible and cap amount of the first layer at the 25% threshold are lower than those at 40% threshold, while the compensation ratios (1–β_1_) and (1–β_2_), at the 25% threshold are higher than those at the 40% threshold. The cap amount of the second layer *CII* model at the two thresholds is the same that is, CNY 421,080. Thus, the final household cumulative *OOP* payments under the model at the two thresholds are calculated in Table 9.

**Table 8:** Indicators of the *CII* model in the *ATP* approach.

**Threshold**	***CD***	**TL_1_**	**TL_2_**	**1-β_1_**	**1-β_2_**
25%	0.25*y*_2_	2.19*y*_2_	421,080	0.91	0.96
40%	0.4*y*_2_	2.27*y*_2_	421,080	0.85	0.93

**Table 9:** Household cumulative *OOP* expenses in the *ATP* approach.

**Threshold**	**Cumulative annual inpatient expenses**	***OOP* expenses**
25%	0.25y_2i_ < *x*_*i*_ ≤ 2.19*y*_2*i*_	0.05*x*_*i*_ + 0.03*y*_2*i*_
	2.19y_2i_ < *x*_*i*_ ≤ 421, 080	0.04*x*_*i*_ − 0.08*y*_2*i*_
	*x*_*i*_ > 421, 080	*x*_*i*_ − 0.08*y*_2*i*_ − 404, 236.80
40%	0.4y_2i_ < *x*_*i*_ ≤ 2.27*y*_2*i*_	0.15*x*_*i*_ + 0.06*y*_2*i*_
	2.27y_2i_ < *x*_*i*_ ≤ 421, 080	0.07*x*_*i*_ − 0.13*y*_2*i*_
	*x*_*i*_ > 421, 080	*x*_*i*_ − 0.13*y*_2*i*_ − 391, 604.40

#### Evaluation of the Performance of the *CII* Model

As can be seen in Table 10, in the case of without any insurance, the incidence of *CHE* at the 25 and 40% thresholds is 20.48 and 17.05%, and the overall severity values at the two thresholds are CNY 3,637.56 and CNY 3,223.75, respectively. In the two-layer *CII* models, all four indicators drop significantly at the two thresholds. The incidence, overall severity, average severity, and regional severity at 25% threshold are 2.07%, CNY 36.33, CNY 9.38, and CNY 1,755.88, respectively, and the corresponding values at the 40% threshold are 2.23%, CNY 56.27, CNY 14.53, and CNY 2,528.98, respectively. Further, whether households or households suffering from *CHE* are insured, the model at the 25% threshold can reduce the incidence and severity of *CHE* to a lower level.

**Table 10:** Performance evaluation of the *CII* model in the *ATP* approach.

		**25%**	**40%**
Without any	*OOP*>*CHE*	1,445	1,203
insurance	incidence	20.48%	17.05%
	Overall severity	3,637.56	3,223.75
	Average severity	939.34	832.48
	Regional severity	17,762.37	18,908.37
*SRBMI*	*OOP*>*CHE*	1,170	910
	Incidence	16.58%	12.90%
	Overall severity	3,165.09	2,699.33
	Average severity	817.34	697.06
	Regional severity	19,087.95	20,930.18
*CII*_1_	*OOP*>*CHE*	414	399
	Incidence	5.87%	5.65%
	Overall severity	1,568.11	1,536.14
	Average severity	404.94	396.68
	Regional severity	26,726.10	27,165.40
*CII*_1_&*CII*_2_	*OOP*>*CHE*	146	157
	Incidence	2.07%	2.23%
	Overall severity	36.33	56.27
	Average severity	9.38	14.53
	Regional severity	1,755.88	2,528.98

#### The Premium Setting in the *CII* Model

Through *MLE*, the scale and shape parameters in the probability distribution function of household non-food expense in 2012 can be calculated, 24,433.57 and 0.83, respectively. The joint probability density function can be expressed as Equation (23). Table 11 presents the premium calculation results.

(23) $$\begin{array}{l} f\left(x,y_{\text{2}}\right)=\frac{1}{\sqrt{2\pi }\times 1.\text{18}\times x}\times \frac{\text{0.83}}{\text{24,433.57}}\\ \times {\left(\frac{y_{\text{2}}}{\text{24,433.57}}\right)}^{-\text{0.17}}e^{-\frac{{\left(\ln\;x-8.\text{89}\right)}^{2}}{2\times 1.\text{1}{\text{8}}^{2}}-{\left(\frac{y_{\text{2}}}{\text{24,433.57}}\right)}^{\text{0.83}}} \end{array}$$

**Table 11:** The *CII* pricing simulation in the *ATP* approach.

	***CII* pricing**	**Pure premium**	**Gross premium**
		**per capita**	**per capita**
25%	Pricing I	344.56	526.97
	Pricing II	516.14	850.11
40%	Pricing I	260.50	398.42
	Pricing II	444.28	731.76

#### The Fund Balance in the *CII* Model

As shown in Table 12, irrespective of the pricing, the total income of the *CII* fund is higher than its total expenditure at the two thresholds. Besides, the surplus of the *CII* fund at the 25% threshold is more than that at the 40% threshold; this means that the 25% threshold can not only ease the households' *CHE* but also keep the fund in a more stable state.

**Table 12:** The fund balance of the *CII* in the *ATP* approach.

**Fund income**			**Fund expenditure**		
***Pricing I***	**25%**	**40%**		**25%**	**40%**
The number of the insured individuals	37,984	37,984	*CII* expenditure	13,236,795	10,110,504
The gross premium per capita	526.97	398.42	The insurance operation management expenses	2,001,655	1,513,348
The total income	20,016,548	15,133,475	The total expenditure	15,238,450	11,623,851
***Pricing II***					
The number of the insured individuals	37,984	37,984	*CII* expenditure	24,463,139	21,111,580
The gross premium per capita	850.11	731.76	The insurance operation management expenses	3,229,054	2,779,529
The total income	32,290,543	27,795,293	The total expenditure	27,692,194	23,891,109

### Comparison

The preceding results show that both approaches can reduce the incidence and severity of household's *CHE*, but the basic approach can alleviate *CHE* more effectively in the two-layer *CII* models. On the one hand, the incidence of *CHE* in the basic approach can be reduced to a low level of 0.85% at the 10% threshold and 0.72% at the 25% threshold; this is clearly <2.07% at the 25% threshold and 2.23% at the 40% threshold in the *ATP* approach. On the other hand, the average and overall severity in the basic approach are also less than those in the *ATP* approach; this means that the basic approach is more effective in reducing the loss for all insured households. However, compared with the two severity indicators mentioned above, the two approaches have different performances in regional severity. Regardless of the thresholds used, the regional severity of *CHE* in the *ATP* approach is lower than that in the basic approach; this means that the *ATP* approach can reduce more effectively the severity for households suffering from *CHE*.

Further, in order to judge the performances of *CII* models fairly, we compare the model based on the basic approach at 10% threshold with that in the *ATP* approach at 25% threshold according to the value of *CHE*; further, we also compare the remaining two models. The conclusions reached about the incidence and severity of the *CHE* are consistent with those in the previous analysis.

In terms of the premium pricing and fund balance of *CII*, there are also some distinctions between the two approaches. The premiums in the basic approach at the 10% threshold, including the pure and gross premiums in the first and second layers, are higher than those in the *ATP* approach at the 25% threshold. The result is similar when the premiums in the basic approach at the 25% threshold are compared with those in the *ATP* approach at the 40% threshold. Besides, from the perspective of the performance of the *CII* fund, the *CII* fund can achieve its financial sustainability irrespective of the threshold used.

## Concluding Remarks

The protection of people from *CHE* has been widely accepted as a desirable objective of global health care financing systems. With the purpose of further reducing *CHE*, the *CII* program in China is being implemented for the past 6 years. However, literature that examines whether different measurements of *CHE* matter in China's *CII* program is scarce. In order to fill this gap, we compare two-layer *CII* models built according to the basic and *ATP* approaches at different thresholds. Exploiting the *CFPS* (2010–2016), we demonstrate that the basic approach is more effective in alleviating the incidence and severity of *CHE* for all insured households, but the *ATP* approach can significantly reduce the severity of *CHE* for households suffering from *CHE*. Further, by comparing two thresholds in the basic approach, it is found that the 25% threshold can bring the incidence and severity of *CHE* for all insured households to a lower level, and the 10% threshold can relieve more effectively the severity of *CHE* for households suffering from *CHE*. However, in the *ATP* approach, the 25% threshold is more effective than the 40% threshold in alleviating the incidence and severity of *CHE*.

Based on the analysis above, the following implications can be drawn. First, regardless of the measurements of *CHE*, the *CII* program can effectively reduce the risk of *CHE*. Therefore, the program should be promoted widely as a supplement to the current *SBMI* system. In addition, to improve the effectiveness of the program, China's *CII* program should be implemented under the basic approach. Further, the threshold used to measure *CHE* should be determined by the goal pursued by the *CII* program. More importantly, as the second-largest economy and the most populous country in the world, China has established a *CII* program that covers a high percentage of *CHE*. Thus, the choice of *CHE* measurement in China's *CII* program can provide valuable guidance for other similar international programs.

However, our research has limitations. First, our two-layer model has been built on China's current healthcare financing system, while its framework prevails around the world. Thus, we believe that possible adjustments are needed according to different countries' circumstance. Second, since China is a large country, different coordination levels are actually adopted based on regional economic and social development. It could be a very interesting direction for our future work.

## Data Availability Statement

The original contributions presented in the study are included in the article/supplementary materials, further inquiries can be directed to the corresponding author.

## Author Contributions

YZ, JV, and DZ conceived and designed the experiments. YG and DH performed the experiments. All authors contributed to the article and approved the submitted version.

## Conflict of Interest

The authors declare that the research was conducted in the absence of any commercial or financial relationships that could be construed as a potential conflict of interest.

## Footnotes

^1^In general, a security surcharge can be set at 10–30% of the pure premium (44). In view of the high medical expense insurance pricing discussed in this paper, we set the ratio at 30%.

^2^The current average operating expenses ratio set by the *PHI* is approximately 20% (individual medical insurance business) or 15% (group medical insurance business). Considering that *CII* involves the *URBMI, NRCM*, and the *PHI* as a group business, the additional premium ratio is set at 15%

^3^The security surcharge of two layers is set to 40% of the pure premium because the coverage is higher, and it is more difficult to obtain medical expenses data that satisfy the actuarial requirements.

^4^The pure premium per capita equals the pure premium divided by the average household population size, and the gross premium per capita equals the gross premium divided by the average household population size.

^5^The compensation ratio is based on the policies of the *SRBMI* in China.
