# Supplementary material for: The Basic vs. Ability-to-Pay Approach: Evidence From China's Critical Illness Insurance on Whether Different Measurements of Catastrophic Health Expenditure Matter
Source: Front Public Health. 2021 Mar 18;9:646810. doi: 10.3389/fpubh.2021.646810 (PMC8044960; doi:10.3389/fpubh.2021.646810)
Supplement: Supplementary file 1 [file Data_Sheet_1.pdf]

Appendix

Table A.1: The comparisons of the basic approach and the *ATP* approach

	The basic approach	The *ATP* approach
Definition of *CHE*	Spending for health care exceeds a certain level of a household's entire budget	Health spending exceeds the threshold of a household's ability to pay for health care
Numerator	*OOP* expenses	*OOP* expenses
Denominator	Total income<br>Total consumption<br>Total expenditure	Household total expenditure minus actual food spending<br>Household total expenditure minus a standard amount representing subsistence food spending<br>Household total expenditure minus a standard amount representing subsistence spending on food, rent and utilities
Advantage	(1) Easy to understand and calculate.<br>(2) Not depend on household allocation decisions across consumption items.	Distinguish between the rich and poor.
Limitation	(1) Fail to distinguish the poor and the rich.<br>(2) Underestimate the poverty.<br>(3) Ignore financing means.	(1) Fail to tell that how far *OOP* expenses eat into resources required for necessities.<br>(2) Ignore financing means.<br>(3) Income data is unavailable in low and middle income countries.

Table A.2: Variable definitions

Variable	Description
Hospitalization	Dummy that equals to one if there is a member of a family being hospitalized in the previous year, otherwise it equals to 0
Ln inpatient medical expenses	The logarithm of household inpatient medical expenses
Agency	The number of hospitals in the province
Bed	The number of beds in the hospitals in the province
Urban	Dummy that equals to one if the individual lives in urban areas, otherwise it equals to 0
Family number	The number of family members
Ln income	The logarithm of the household income
Insurance	The number of people in a family with basic social medical insurance
Age	The age of the family financial respondent
Marriage status	The marriage status of the financial respondent was divided into single, divorced, married, widowed and cohabitation
Health condition	The health condition of the financial respondent is self-assessed on a five-point scale, ranging from "The worst" (−2) to "The best" (+2)

Table A.3: Data description (2010)

Variable	Obs	Mean	Std. Dev.	Min	Max
Hospitalization	7,579	0.1735057	0.3787089	0	1
Inpatient medical expenses	7,579	1,642.721	7,109.463	0	200,000
Agency	7,579	874.4276	343.7654	277	1377
Bed (Ten thousands)	7,579	15.30977	5.603493	4.04	25.58
Urban	7,579	0.5515239	0.497371	0	1
Family number	7,579	3.50376	1.569732	1	15
Ln income	7,579	9.958263	1.199798	0	13.60726
Insurance	7,579	1.964903	1.277155	0	10
Age	7,579	50.72714	12.8005	17	97
Marriage status					
Single	171				
Divorced	192				
Married	6,682				
Widowed	504				
Cohabitation	30				
Health condition					
-2	159				
-1	494				
0	643				
1	3,009				
2	3,274				

Table A.4: Data description (2014)

Variable	Obs	Mean	Std. Dev.	Min	Max
Hospitalization	10,080	0.230754	0.4213362	0	1
Inpatient medical expenses	10,080	3,404.887	14,328.4	0	520,000
Ln inpatient medical expenses	2,326	8.828464	1.210692	5.010635	13.16158
Agency	10,080	1,021.303	438.6881	161	1854
Bed (Ten thousands)	10,080	20.90744	9.254966	3	36
Urban	10,080	0.4837302	0.49976	0	1
Family number	10,080	3.768254	1.796432	1	17
Ln income	10,080	10.45733	1.001489	0	15.26726
Insurance	10,080	2.1375	1.182496	0	9
Age	10,080	49.69902	13.97626	16	92
Marriage status					
Single	447				
Divorced	239				
Married	8,680				
Widowed	662				
Cohabitation	52				
Health condition					
-2	1,797				
-1	1,621				
0	3,750				
1	1,712				
2	1,200				

Table A.5: Estimates of the regression models (2014)

	Hospitalization	Ln inpatient medical expenses
**Agency**		-0.0004446***
		（0.0001223）
**Bed**		0.0182864***
		（0.0059627）
**Urban**	0.1811973 ***	0.1920981***
	（0.0529236）	（0.0541036）
**Family number**	0.0277184	-0.0362305**
	（0.0172121）	（0.0181957）
**Ln income**	-0.0649381 **	0.1207951**
	（0.0283993）	（0.0290366）
**Insurance**	0.3779734 ***	0.0736707***
	（0.0251204）	（0.0261489）
**Age**	0.0162383 ***	0.0111022***
	（0.0021592）	（0.0019725）
**Marriage status**		
Divorced	0.4853371 **	-0.0426169
	（0.2226117）	（0.2411069）
Married	0.1899185	-0.0559939
	（0.1599719）	（0.1809888）
Widowed	-0.1830898	-0.4033614*
	（0.191195）	（0.2123892）
Cohabitation	0.0257	-0.3493461
	（0.4208557）	（0.3956593）
**Health condition**		
2	-0.3068183***	-0.0018289
	（0.0901855）	（0.0899121）
1	-0.4184451***	0.1342498
	（0.0812338）	（0.0864674）
-1	0.0669768	0.1106135
	（0.0723347）	（0.071394）
-2	0.7321299***	0.2965411***
	（0.0668217）	（0.0646634）
**C**	-2.626715***	6.886184***
	（0.3429847）	（0.3545954）
